# Pim-1 acts as an oncogene in human salivary gland adenoid cystic carcinoma

**DOI:** 10.1186/s13046-014-0114-5

**Published:** 2014-12-31

**Authors:** Xin Zhu, Jia-jie Xu, Si-si Hu, Jian-guo Feng, Lie-hao Jiang, Xiu-xiu Hou, Jun Cao, Jing Han, Zhi-qiang Ling, Ming-hua Ge

**Affiliations:** Zhejiang Cancer Research Institute, Zhejiang Cancer Hospital, 310022 Hangzhou, China; Department of Head and Neck Surgery, Zhejiang Cancer Hospital, 310022 Hangzhou, China

**Keywords:** Salivary gland adenoid cystic carcinoma, Pim-1, RUNX3

## Abstract

**Background:**

Pim-1 (Provirus integration site for Moloney murine leukemia virus 1) belongs to the Ser/Thr kinase family and plays a pivotal role in occurrence and development of oncogenesis. Recent studies have demonstrated that Pim-1 phosphorylates RUNX3 and alters its subcellular localization. However, few studies have concerned the implications of Pim-1 in the salivary gland adenoid cystic carcinoma (ACC). In this study, we aimed to clarify the function of Pim-1 in ACC in vitro. Meanwhile, we measured the levels of Pim-1 and RUNX3 in the ACC tissues. The correlations between Pim-1/RUNX3 levels and clinical parameters were also analyzed.

**Methods:**

SACC-83 and SACC-LM cells were transfected with the Pim-1 siRNA. Pim-1 mRNA and protein expression were measured using real-time PCR and immnuoblot, respectively. Cell proliferation was analyzed by CCK-8 assay. Cell cycle, apoptosis, and mitochondrial membrane potential were detected by flow cytometry. Effects of Pim-1 on cells’ invasion were evaluated by transwell migration assay. Pim-1 and RUNX3 levels in ACC tissues were examined by immunohistochemistry.

**Results:**

Pim-1 siRNA reduces cell proliferation, induces apoptosis, causes cell cycle arrest through cell cycle related proteins (Cyclin D1 and CDK4), mitochondrial depolarization, and decreases invasive ability in SACC-83 and SACC-LM cells. Pim-1 and RUNX3 levels are significantly relevant and associated with T-stage and nerve invasion in the ACC tissues.

**Conclusions:**

This study demonstrates the oncogenic role of Pim-1 in ACC. The findings also suggest that Pim-1 may serve as a neoteric therapeutic target and potential prognostic marker for ACC cancer.

## Background

Salivary gland adenoid cystic carcinoma (ACC) accounts for approximately 10% of all epithelial salivary tumors. Clinical characteristics of ACC include high aggressive to nerve and vessel, high rate of recurrence, and frequent metastasis to lung. The 5-year survival rate of patients with highly metastatic ACC is less than 20%. Since ACC is not sensitive to radiotherapy or chemotherapy, surgical resection is the most common treatment for ACC. Like most other tumors, the interaction of oncogenes and tumor suppressor genes is involved in the development of ACC. However, the precise mechanism responsible for its oncogenesis is not completely understood [1-3]. Therefore, it is warranted to study the molecular mechanism of salivary gland ACC to gain insight into its diagnosis, prognosis, and treatment.

As a widely accepted tumor suppressor gene, RUNX3 (human runt-related transcription factor 3) functions in major physiological and pathological processes. Many reports have shown essential behavior of RUNX3 in a variety of cancers [4]. Our previous study presented the expression of RUNX3 in normal salivary glands and salivary ACC. Moreover, the expression of RUNX3 is obviously correlated with histopathological growth pattern, T stage, distant metastasis, and patient’s survival. These results suggested that the low level of RUNX3 in salivary gland ACC might play a key role in tumor progression and have prognostic value in ACC [5]. Subsequently, we found that the RUNX3 mislocalization was related to the progression of the ACC by laser scanning confocal microscope [6].

Recent studies have demonstrated that Pim-1, which acted as an oncogene, could phosphorylate RUNX3 and alter its subcellular localization [7,8]. As belongs to the Ser/Thr kinase family, Pim kinases can phosphorylate a large range of cellular substrates to exert their physiological activities, which are involved in cell differentiation, cell proliferation, cell cycle and apoptosis. Pim-1 plays a pivotal role in tumorigenesis and overexpression of it has been implicated in several cancers [9,10]. Collectively, these reports suggest that Pim-1’s function is important in the progression of cancer.

However, the oncogenic role of Pim-1 in ACC has not yet been examined. As an effective tool to achieve gene silence, small interfering RNAs (siRNAs) were used in lots of researches to clarify the gene function [11-13]. In this study, using siRNA transfection in vitro, we aim to clarify the gene function of Pim-1 in ACC through the detection of cell proliferation, cell cycle, cell apoptosis and cell invasion. Meanwhile, a Pim-1 inhibitor (SGI-1776) was used to confirm the affection of Pim-1 on the cell proliferation ability. The RUNX3, Cyclin D1 and CDK4 (cyclin-dependent kinase 4) expression after Pim-1 siRNA transfection was investigated as well. Furthermore, the relationship between Pim-1 and RUNX3 was deduced in the ACC tissues. The correlation of Pim-1/RUNX3 and clinical parameters were analyzed. This study provided new data of Pim-1 activity in ACC and is suggestive that Pim-1 has potential to become a tumor marker as well as a therapy target of cancer.

## Materials and methods

### Tissue specimens

Fifty-four patients with histopathologically proven salivary gland adenoid cystic carcinoma (ACC) in Zhejiang Cancer Hospital between July 2006 and July 2010 were included for this study. The study was approved by the Ethics Committee of Zhejiang Cancer Hospital, and patients have signed informed consent.

### Cell culture and siRNA transfection

SACC-83 and SACC-LM were kind gifts from Prof. Li Shengling (Peking University School of Stomatology). SACC-83 is derived from human ACC tissue and SACC-LM is a lung metastasis cell line of SACC-83 [14,15]. It is considered that SACC-LM is more malignant then SACC-83. Both cell lines were cultured in RPMI-1640 (Gibco, USA) supplemented with 10% fetal bovine serum (FBS) (HyClone Laboratories, Logan, USA) in a humidified atmosphere of 5% CO2 at 37°C. Pim-1 siRNA and control siRNA were obtained from Santa Cruz Company (CA, USA). SACC-83 and SACC-LM cells were seeded in a 96-well culture plate with a density of 5 × 10^3^ cells/well or in a 6-well culture plate with a density of 1 × 10^6^ cells/well. After 24 h, siRNAs (0.1 μmol/L) were transfected with Oligofectamine TM Reagent (Invitrogen, Carlsbad, CA, USA) into the cells according to manufacturer's protocol.

### SGI-1776 treatment

SGI-1776 (Selleckchem, Houston, TX, USA) was dissolved in DMSO to obtain a stock solution at 10 mmol/L and stored at −80°C. After SACC-83 and SACC-LM were cultured, SGI-1776 was added to the culture system to achieve the final doses of 0–10 μmol/L. The highest concentration of DMSO in the culture was 0.1% and we tested that it had no effect on the cells viability.

### Cell proliferation assay

According to manufacturer's instructions, Cell Counting Kit-8 (CCK-8) was used to determine the cell proliferation (Dojindo Biotechnology, China). Briefly, SACC-83 and SACC-LM cells were seeded in a 96-well cell plate for 24 h, and then transfected with Pim-1 siRNA/control siRNA, for 48 and 72 h and exposed to SGI-1776 for 24, 48 and 72 h respectively. Then the medium were discarded and replaced with 100 μl of fresh medium containing 10% CCK-8. After cells were incubated at 37°C for 4 h, the absorbance was detected at 450 nm on a microplate reader.

### Colony formation assay

After transfected with Pim-1 siRNA and control siRNA for 72 h, SACC-83 and SACC-LM cells were washed with PBS (Phosphate Buffer Solution) and trypsined. Then the cells were seeded into a 12-well plate with a density of 500 cells/ well. After 7 days of incubation, colonies were stained by 0.5% crystal violet (Sigma-Aldrich, St. Louis, MO, USA) and counted directly.

### Evaluation of live cell undergoing apoptosis

In this study, Annexin V-FITC and propidium iodide (PI) (BD Biosciences, USA) were used to distinguish intact, dead and apoptotic cells by using the flow cytometric method. SACC-83 and SACC-LM cells were harvested and washed with cold PBS after transfected with Pim-1 siRNA and control siRNA for 72 h. Subsequently, the cells were resuspended in 100 μL binding buffer. 5 μL Annexin V-FITC and 1 μL PI were added to the cell suspension and incubated in darkness at room temperature for 15 min. Thereafter, 400 μl binding buffer was added to each sample and then the cells were analyzed by using the flow cytometer (BDLSR, Becton Dickinson, USA).

### Cell cycle detection

Cell cycle assay was performed using the Cycle Test Plus™ DNA Reagent Kit (340242, Becton Dickinson, USA) following the manufacturer’s instructions. SACC-83 and SACC-LM cells were harvested and washed with cold PBS after transfected with Pim-1 siRNA and control siRNA for 72 h. Then, the cells were fixed in pre-cooled 70% ethanol for 24 h at 4°C. After that, the cells were dyed with PI and detected by flow cytometry (BDLSR, Becton Dickinson, USA) to evaluate the cell cycle distribution.

### Transwell chamber invasive assay

After transfected with Pim-1 siRNA and control siRNA for 72 h, SACC-83 and SACC-LM cells were obtained and plated at 1.0 × 10^5^ cells/well in 0.5 mL of serum-free medium in 24-well matrigel-coated transwell units with polycarbonate filters (8 μm pore size; Costar Inc., Milpitas, CA, USA). The outer chambers were filled with 0.5 mL of RPMI 1640 medium supplemented with 10% FBS. After incubated for 24 h, the cells were fixed in methanol and stained with 2% crystal violet. The top surface of the membrane was gently removed and the invading cells were counted in five randomly selected fields under a microscope.

### Assessment of mitochondrial membrane potential

The mitochondrial membrane potential (MMP), which is recognized as a typical marker of early apoptosis was measured by the fluorescent probe JC-1 (MultiSciences Company, Hangzhou, China) in this study. As a cationic and lipophilic dye, JC-1 presents a fluorescence emission shift from green (525 nm) to red (590 nm) to indicate the potential-dependent accumulation in mitochondria. In healthy and normal cells with low membrane potentials, JC-1 appears as a monomer and produces a green fluorescence. At high membrane potentials, J aggregates is induced by JC-1 and the red fluorescence was emerged. In accordance with the manufacturer's protocol, after SACC-83 and SACC-LM cells were transfected by the Pim-1 siRNA for 72 h, the cells were harvested, centrifuged and re-suspended in 1 ml staining buffer. After stained with 1ul JC-1 staining solutions, the cells were incubated at 37°C in the dark for 30 min. Subsequently, the cells were washed twice with warm PBS buffer and re-suspended in 0.5 ml PBS buffer. Flow cytometry was performed to examine the red/green fluorescent signals.

### Quantitative real-time reverse transcription-PCR

SACC-83 and SACC-LM cells were transfected with Pim-1 siRNA or control siRNA for 72 h. Total RNA was extracted by using Trizol (Invitrogen, Carlsbad, CA, USA) and reverse-transcription was done with PrimeScipt™ RT reagent Kit (TAKARA BIO INC., Otsu, Shiga, Japan) according to manufacturer’s instructions. Quantitative real-time PCR (qRT-PCR) was performed on an ABI Prism 7500 instrument (Applied Biosystems, Foster city, CA, USA) by using the SYBR Premix Ex Taq (TAKARA BIO INC., Dalian, China). Pim-1 and GAPDH mRNA levels were measured by their specific primers:

Pim-1 (F:CTGCTCAAGGACACCGTCTACA;

R: GATGGTAGCGGATCCACTCTG);

GAPDH (F: 5'-GAAGGTGAAGGTCGGAGTC-3';

R: 5'- GAAGATGGTGATGGGATTTC-3')

After PCR amplification, the dissociation of SYBR Green-labeled cDNA was carried out to affirm that there were no nonspecific PCR products. 2^-∆∆Ct^ method was performed to analyze the relative quantification of Pim-1 expression.

### Western blot analysis

SACC-83 and SACC-LM cells were trypsinized and washed with cold PBS after transfected with Pim-1 siRNA or control siRNA for 72 h. Then cell pellet was lysed with RIPA Lysis Buffer (Beyotime, Nanjing, China). ACC tissue samples were ground with the Tissue Lyser-II (Qiagen, Germany) and lysed with RIPA Lysis Buffer. Protein concentrations were determined by the BCA Protein Assay Kit (Beyotime, Nanjing, China). All samples were stored at −70°C prior to electrophoresis. Each sample containing 50 μg of protein were subjected to 12% SDS-PAGE and separated by electrophoresis. Then the proteins were transferred electrophoretically from the gel to nitrocellulose membrane (Immobilon-P^SQ^ Transfer Membrane, Millipore Corporation, Bedford, U.S.A.). To prevent non-specific binding of reagents, the membranes were blocked in TBS buffer (50 mM Tris-Cl, 150 mM NaCl, pH 7.6) containing 5% non-fat dry milk at room temperature for 3 h. Then blotted with primary antibodies of Pim-1 (Novus biologicals, Cambridge, UK) (1:1000 dilution), RUNX3 (Abgent, San Diego, CA, USA) (1:100 dilution), Cyclin D1 (Proteintech, Whhan, China) (1:1000 dilution), CDK4 (Proteintech, Whhan, China) (1:1000 dilution), β-actin (Abcam, New Territories, HK) (1:200 dilution) and GAPDH (Huabio Technology, Hangzhou, China) (1:2000 dilution) at 4°C overnight and developed with peroxidase-labeled secondary antibodies (Huabio Technology, Hangzhou, China). After that, the membranes were extensive washed in TBST and exposed to 2 mL ECL chemiluminescence reagent. The images were captured and analyzed on the Bio-Rad GelDoc XR.

### Immunohistochemistry

Section (4 μm) of paraffin-embedded tissues were cut, mounted on glass slides (MS-coated glass, Mats-unami, Oaska, Japan), and dried overnight at 37°C. After deparaffinization, antigen retrieval in 0.01 M citrate buffer, and inactivation of endogenous peroxidase activity in 3% H_2_O_2_/methanol, the slides were incubated with antibody for Pim-1 (Novus biologicals, Cambridge, UK) (1:200 dilution) and RUNX3 (Abcam, New Territories, HK) (1:200 dilution) at 4°C overnight. The immunoreactivity was visualized using a streptavidin–biotin peroxidase staining kit (Histofine Simple Stain Max PO Multi, Nichirei, Tokyo, Japan) and DAB solution (Simple Stain DAB, Nichirei). The results were presented as percentage of nucleus staining positive cells and the total cells. The scores of staining results were given as negative and positive. The criterion was consulted our previous study [16].

### Statistical analysis

All data were analyzed using SPSS 10.0 software. Results from in vitro experiments were expressed as mean ± standard deviations and statistically analyzed by the one-way analysis variance (ANOVA). Associations between Pim-1, RUNX3 levels and clinicopathologic parameters were analyzed using the X^2^ test or the Fisher exact test. Survival analysis was carried out by the Kaplan–Meier method and significant differences were assessed by means of the log-rank test. *P* values < 0.05 were considered to be statistical significance.

## Results

### Effect of the Pim-1 siRNA on the Pim-1 level

**Figure 1:**
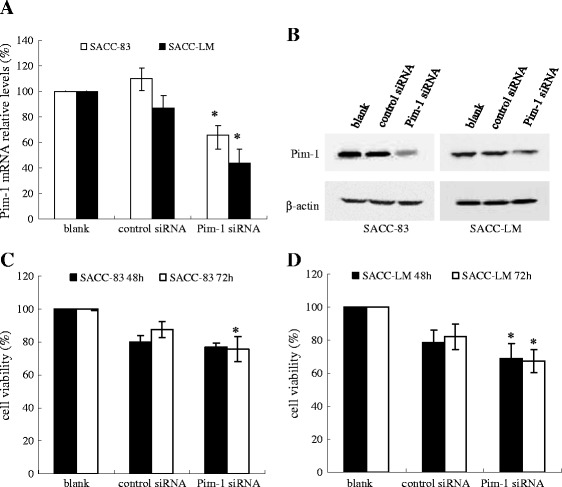
**Effect of Pim-1 siRNA on Pim-1 expression level and cell viability in SACC cells. A**. Real time PCR results displayed the Pim-1 mRNA expression in both cell lines. **B**. Western blot showed the inhibition effect of Pim-1 siRNA on Pim-1 protein levels in both cell lines. The expression of β-actin was used as a quantitative control. **C**. CCK-8 assay results of SACC-83 cell after transfected with Pim-1 siRNA for 48 h and 72 h. **D**. CCK-8 assay results of SACC-LM cell after transfected with Pim-1 siRNA for 48 h and 72 h. Data were showed as mean ± SD. p < 0.05, *Pim-1 siRNA group compared with control siRNA group.

SACC-83 and SACC-LM cells were divided into three groups including blank, control siRNA, and Pim-1 siRNA groups. After siRNA transfection, Pim-1 transcript and protein levels were measured by real-time PCR and Western blot, respectively. Pim-1 transcript (Figure 1A) and protein levels (Figure 1B) were significantly decreased after Pim-1 siRNA transfection compared to those of blank and control siRNA, indicating that Pim-1 siRNA effectively inhibited Pim-1 expression in both cell lines.

### Effect of Pim-1 RNAi on cell proliferation in SACC-83 and SACC-LM

**Figure 2:**
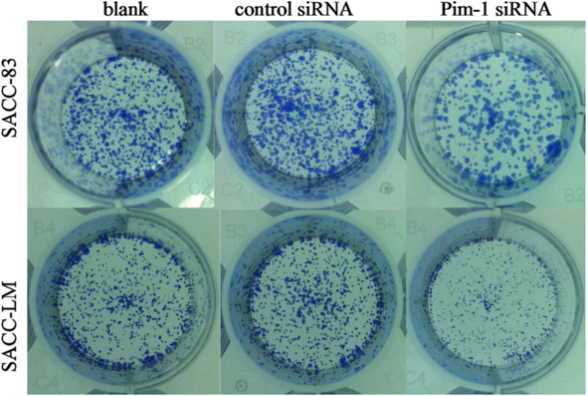
**Clonogenic assay of SACC cells transfected with Pim-1 and control siRNA for 72 h.** Cells were cultured for 10 days and stained with crystal violet in 12 well plate.

After transfection with Pim-1 or control siRNA for 48 h and 72 h, cell viabilities were determined by using the CCK-8 assay. Results represented that viabilities of both cells showed a decreased trend along blank, control siRNA and Pim-1 siRNA. Moreover, there were significant differences between the control siRNA and Pim-1 siRNA groups in the SACC-LM cell (Figure 1C and D). Meanwhile, the colony formation for both SACC cell lines after transfected with Pim-1 siRNA was evaluated. As shown in Figure 2, colonies in SACC-83 and SACC-LM cells which tranfected with Pim-1 siRNA were significant reduction compared to those of blank and control siRNA. These suggested that Pim-1 siRNA could reduce the proliferation activity in SACC cells.

### Effect of SGI-1776 on cell proliferation in SACC-83 and SACC-LM

**Figure 3:**
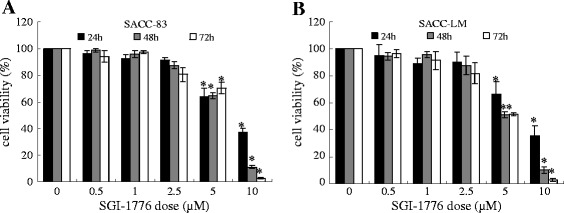
**Effect of SGI-1776 on cell viability in SACC-83 and SACC-LM cells. A**. CCK-8 assay results of SACC-83 cell after exposed to 0–10 μmol/L SGI-1776 for 24 h, 48 h and 72 h. **B**. CCK-8 assay results of SACC-LM cell after exposed to 0–10 μmol/L SGI-1776 for 24 h, 48 h and 72 h. Data were showed as mean ± SD. p < 0.05, *0.5-10 μmol/L SGI-1776 groups compared with 0 μmol/L SGI-1776 group.

Figure 3 showed that after exposed to 0, 0.5, 1, 2.5, 5 μmol/L SGI-1776 for 24 h, 48 h and 72 h, the proliferation rates of SACC cells were decreased in a dose and time-dependent manner. In both cells, there are significant differences between the 5, 10 μmol/L groups and control groups at all the SGI-1776 treatment time points.

### Effect of the Pim-1 siRNA on the apoptosis

**Figure 4:**
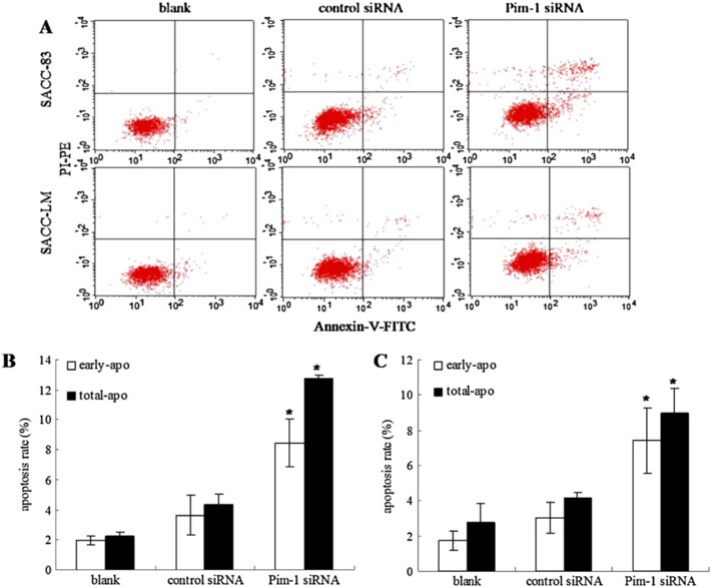
**Apoptosis induced by Pim-1 siRNA in SACC cells. A**. Contour diagram of Annexin-V/PI flow cytometric evaluation of apoptosis after Pim-1 and control siRNA transfection for 72 h. **B**. Early-apoptosis and total-apoptosis rates of SACC-83 induced by Pim-1 siRNA. **C**. Early-apoptosis and total-apoptosis rates of SACC-LM induced by Pim-1 siRNA. Results were shown as mean ± SD. p < 0.05, *Pim-1 siRNA group compared with control siRNA group.

In this study, both early-apoptosis rate (Annexin-V+/PI-) and total apoptosis rate (Annexin-V+/PI- and Annexin-V+/PI+) were analyzed to describe the effect of Pim-1 knockdown on apoptosis. Flow cytometry analysis showed that a majority of cells in the blank group were intact live cells. After siRNA transfection, apoptosis and dead cells increased measureably (Figure 4A). The data demonstrated that both SACC-83 and SACC-LM cells showed a significant increase in early-apoptosis rate in Pim-1 siRNA group, compared to control siRNA group after 72 h transfection (Figure 4B and C). A consistent trend of total cell apoptosis rates was also observed in this study. These data demonstrate that Pim-1 siRNA can dramatically induce apoptosis in SACC cells.

### Effect of the Pim-1 siRNA on cells cycle

**Figure 5:**
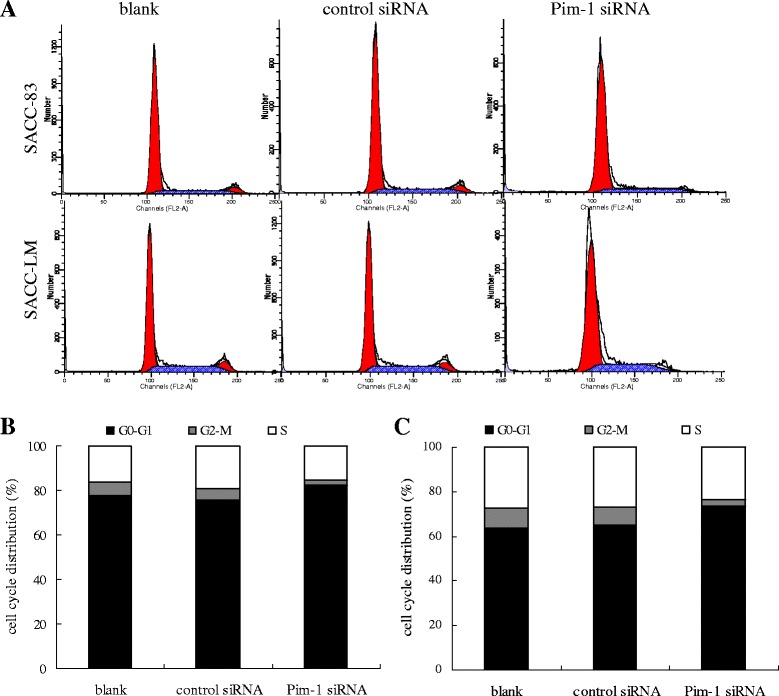
**Effect of Pim-1 siRNA on cell cycle of SACC cells. A**. Cell cycle rate measured by FCM analysis after PI staining. **B**. Cell cycle distribution of SACC-83 cells after Pim-1 siRNA transfection. **C**. Cell cycle distribution of SACC-LM cells after Pim-1 siRNA transfection.

After transfection with Pim-1 or control siRNA for 72 h, cell cycles were determined by flow cytometer (Figure 5A). In SACC-LM cells, the percentage of G0-G1 phase in Pim-1 siRNA transfected cells was significantly higher than that in control siRNA transfected cells (73.52% *vs* 64.99%, *p* < 0.05), whereas the percentages of G2-M phase and S phase in Pim-1 siRNA transfected cells were significantly lower than those in control siRNA transfected cells (2.86% *vs* 8.09%, *p* < 0.05; 23.62% *vs* 26.91%, *p* < 0.05) (Figure 5C). The same tendency was observed in SACC-83 cells except that there was no significant difference in the percentage of S phase between Pim-1 siRNA and control siRNA groups (Figure 5B). The results indicated that the down-regulation of Pim-1 led to cell cycle arrest and reduced the proliferation of SACC cells, which corroborates results from the CCK-8 assay.

### Effect of the Pim-1 siRNA on cell invasion

**Figure 6:**
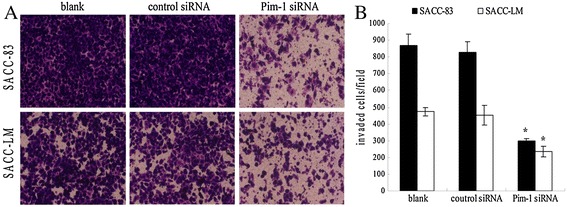
**Suppressive effect of Pim-1 siRNA on the cell invasion in SACC cells. A**. Crystal violet staining images of invasive SACC-83 and SACC-LM cells after Pim-1 siRNA transfection for 72 h. **B**. Quantification of the number of invaded SACC-83/SACC-LM in control siRNA and Pim-1 siRNA groups, respectively. Results were shownas mean ± SD. p < 0.05, *Pim-1 siRNA group compared with control siRNA group.

Figure 6 showed that in both SACC-83 and SACC-LM cells, the invasion potentials in blank and control siRNA were not significantly different, while the invasion potential of the Pim-1 siRNA transfected cells were significantly decreased compared to the control group. In contrast, the invasion inhibitory effect by Pim-1 siRNA was more obvious in SACC-83 cells than in SACC-LM cells.

### Effect of the Pim-1 siRNA on the mitochondrial function

**Figure 7:**
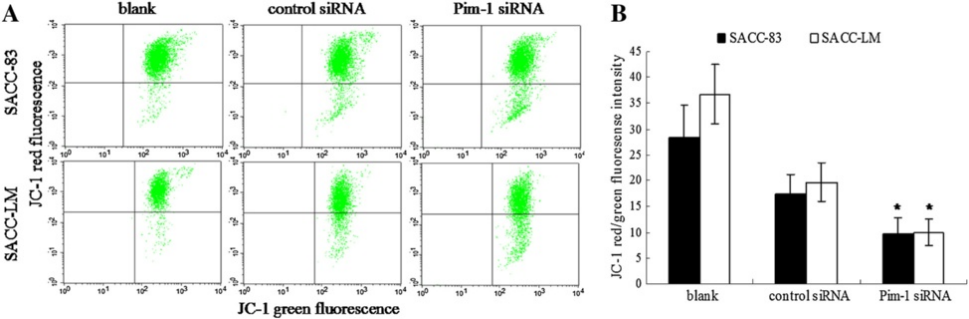
**Depolarization of mitochondria membrane induced by Pim-1 siRNA in SACC cells. A**. Cells were stained with JC-1 and analyzed by cytometer after Pim-1 and control siRNA transfection for 72 h. **B**. Mitochondrial depolarization presented by decrease in the red/green fluorescence intensity ratio. Results were showed as mean ± SD. p < 0.05, *control siRNA group compared with Pim-1 siRNA group.

After transfection by Pim-1 siRNA in both SACC cells, the JC-1 red fluorescence was reduced while JC-1 green fluorescence was increased relative to control siRNA (Figure 7A). Therefore, the data presented a significant decrease in the red/green fluorescence intensity ratio, indicating a decreased ΔΨm (Figure 7B). This result suggests that the reduction of Pim-1 expression could drive mitochondrial depolarization.

### RUNX3, Cyclin D1 and CDK4 expression of SACC cells after Pim-1 siRNA transfection

**Figure 8:**
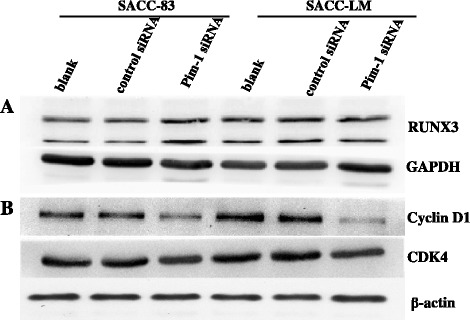
**Pim-1 depletion altered the RUNX3, Cyclin D1 and CDK4 levels. A**. Western blot analysis showed the expression of RUNX3 protein in SACC cells after Pim-1 siRNA transfection. The expression of GAPDH was used as a loading control. **B**. Western blot analysis showed the expression Cyclin D1 and CDK4 protein in SACC cells after Pim-1 siRNA transfection. The expression of β-actin was used as a loading control.

After transfection with Pim-1 siRNA for 72 h, RUNX3, Cyclin D1 and CDK4 protein expression of both SACC cells were measured by Western blot. It was showed that there was a slight increase in RUNX3 level in SACC-83 cell (Figure 8A). Figure 8B illustrated that Pim-1 knockdown may downregulate the Cyclin D1 and CDK4 protein expression and contribute to the cellular effects we identify in this study.

### Correlation between Pim-1 and RUNX3 protein levels in ACC tissues

**Figure 9:**
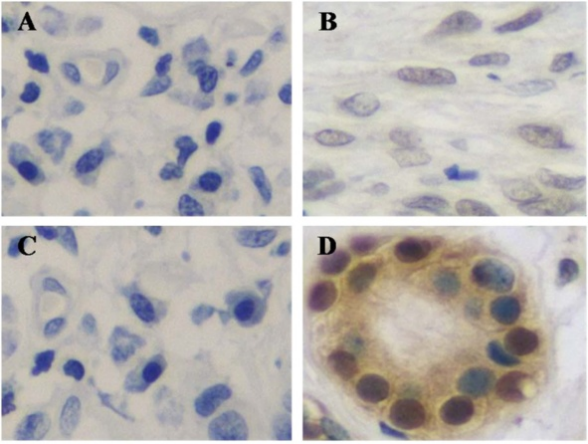
**Immunohistochemical (IHC) staining of Pim-1 and RUNX3 in ACC. A**. Negative Pim-IHC staining in ACC. **B**. Positive Pim-1 IHC staining in ACC. **C**. Negative RUNX3 IHC staining in ACC. **D**. Positive RUNX3 IHC staining in ACC. Magnificant factor: ×400.

Immunohistochemical (IHC) staining of Pim-1 and RUNX3 in ACC tissues is shown in Figure 9. Positive ratios of Pim-1 and RUNX3 were 83.33% (45/54) and 20.37% (11/54), respectively. Table 1 showed there is a significant inverse correlation between the expression of Pim-1 and RUNX3.

**Table 1:** **Relationship between the level of Pim-1 and RUNX3**

**Variable**	**Patients (total = 54)**		***p*** **-value**
	**Negative**	**Positive**	
Pim-1	9	45	
RUNX3	43	11	0.003

### Correlation between the level of Pim-1 and RUNX3 and the clinical characteristics in ACC tissues

To identify the role of Pim-1 and RUNX3 expression in ACC, we analyzed the correlation between Pim-1 and RUNX3 expression in ACC tissues and clinicopathologic indexes. The IHC staining results are displayed in Figure 7. Table 2 illustrates that both Pim-1 and RUNX3 levels were closely associated with T-status and nerve invasion (*p* < 0.05), but not with gender, age, tumor location, histological grade type, lymph node involvement or distant metastasis (*p >* 0.05). It can be deduced that patients with more malignant tumors tend to have higher Pim-1 and lower RUNX3 level.

**Table 2:** **Relationship between the Pim-1, RUNX3 level and the clinical characteristics**

**Variable**	**Patients (total = 54)**		**Pim-1**		***p*** **-value**	**RUNX3**		***p*** **-value**
			**Negative**	**Positive**		**Negative**	**Positive**	
Gender	Male	23	4	19	1.000	19	4	0.741
	Femal	31	5	26		24	7	
Age	<46	26	3	23	0.495	21	5	1.000
	≥46	28	6	22		22	6	
T-status	T1-2	22	8	14	0.002	14	8	0.036
	T3-4	32	1	31		29	3	
Tumor location	Major salivary	21	4	17	0.723	15	6	0.305
	Minor salivary	33	5	28		28	5	
Histological type	Cribiform	24	4	20	1.000	22	2	0.116
	Tubula	12	2	10		8	4	
	Solid	18	3	15		13	5	
Lymph node involvement	Yes	9	0	9	0.328	8	1	0.667
	No	45	9	36		35	10	
Nerve invasion	Yes	0	1	29	0.007	28	2	0.007
	No	24	8	16		15	9	
Distant metastasis	Yes	8	0	8	0.326	8	0	0.184
	No	46	9	37		35	11	

### Survival analysis

**Figure 10:**
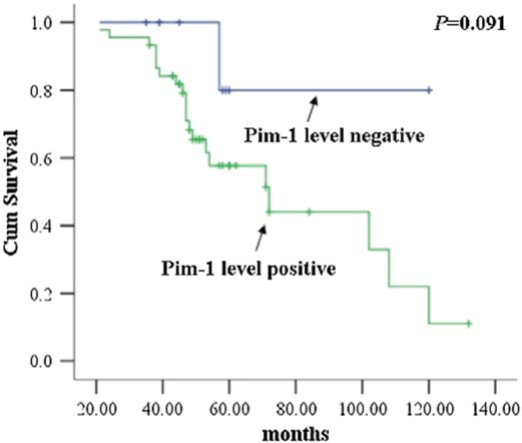
**Kaplan–Meier curve for the relationship between Pim-1 protein expression which analyzed by immunohistochemistry (IHC) and survival time of ACC patients.**

Kaplan–Meier survival curves (Figure 10) showed that Pim-1 level had a weak association with overall survival of ACC patients (*p* = 0.091). Patients with lower Pim-1 levels had a better outcome than that with higher Pim-1 levels.

## Discussion

Increasing evidence shows the important role of Pim-1 in many cancers. Various investigations have linked Pim-1 to aggressive malignant behavior and poor clinical outcome in many cancer cells, including gastric cancer [17], prostate cancer [18], esophageal squamous cell carcinoma [19], breast cancer [20], lung cancer [21], colon cancer [22], and hematological cancer [23]. However, few studies has concern the implications of Pim-1 in salivary ACC.

In this study, SACC-83 and SACC-LM cell lines were used to evaluate the function of Pim-1 in salivary ACC. After Pim-1 siRNA transfection, the mRNA and protein levels of Pim-1 were significantly decreased in both cell lines, suggesting that Pim-1 siRNA inhibits endogenous Pim-1 expression. CCK-8 assay and colony formation results revealed that down-regulation of Pim-1 could restrain the cell viability of SACC-83 and SACC-LM. Meanwhile, we use SGI-1776, which was confirmed as a novel inhibitor of the PIM kinases to test the function of Pim-1. SGI-1776 is an imidazo [1,2-b] pyridazine compound and have been proved to effectively reduced Pim-1 activity in several researches [24-26]. Our results displayed that SGI-1776 could evidently induced the SACC cells growth inhibition. It could be deduced from these results that Pim-1 is important for cell proliferation in ACC cells.

To disarrange cell cycle progression is one of the central features of malignant cancer cells. In the present study, it was found that G0-G1 phase cells were increased while G2-M phase and S phase cells were decreased after Pim-1 siRNA transfection. Furthermore, we observed the down-regulation of Cyclin D1 and CDK4 protein expression. Cyclin D1 and CDK4 work together to form the complex and to promote G1 phase progression and regulate the cell cycle G1/S transition [27]. Those data demonstrates that the G0/G1 cell cycle arrest was induced by knocking down Pim-1 and probably mediated by Cyclin D1 and CDK4. Annexin V-FITC/PI and transwell assay results showed that the apoptosis rates were dramatically ascended and the invasion ability was significantly reduced after Pim-1 siRNA transfection in both cell lines. Meanwhile, the mitochondrial dysfunction indicated by membrane potential decrease after siRNA transfection in both cells was investigated. These findings reinstates the oncogenic function of Pim-1 in ACC cell lines, indicating the important role of Pim-1 in tumorigenesis of ACC.

Meanwhile, the transwell migration results show that Pim-1 siRNA could dramatically reduce the invasion capacity of SACC cells. The invasion inhibition effect by the Pim-1 siRNA was stronger in the SACC-83 cells than in the SACC-LM cells. The immunohistochemical results show that Pim-1 level is significantly associated with nerve invasion in ACC patients. The findings suggest Pim-1 expression may be a critical marker for ACC invasion.

In 2006, Aho *et al.* found that the C-terminal part of human RUNX3 associates with Pim-1 by using the yeast two-hybrid system [8]. Subsequently, Kim *et al.* demonstrated that Pim-1 phosphorylates four Ser/Thr residues within the Runt domain and stabilizes RUNX3 protein [7]. In SACC-83, we observed an increase of RUNX3 protein level after Pim-1 transfection. In ACC tissues, there was a significant reverse correlation between the Pim-1 and RUNX3 expression by IHC evaluation.

Moreover, we investigated the Pim-1 and RUNX3 in ACC tissues. The IHC results show that both Pim-1 and RUNX3 levels were significantly associated with T stage and nerve invasion. Patients with advanced T stage and nerve invasion had a higher Pim-1 and lower RUNX3 level. High expression of Pim-1 and low expression of RUNX3 were associated with aggressive tumor behavior. This evidence suggests an importance of the interaction between Pim-1 and RUNX3 in ACC. As to other clinical features such as gender, age, tumor location, histological grade type, lymph node involvement or distant metastasis, we did not found significant associations between the Pim-1/RUNX3 and them, which might owing to the limitation of the cancer quantity and need to be further studied.

Survival analysis indicated that Pim-1 level had a weak association with overall survival of the ACC patients (*p* = 0.091). Patients with lower Pim-1 level had a better outcome than that with higher Pim-1 level. Choi *et al.* found that Pim-1 expression might be used as a possible prognostic factor in laryngeal squamous cell carcinoma [28]. Peng *et al.* presented that expression of Pim-1 in tumors, tumor stroma, and tumor-adjacent mucosa could indicate the prognosis of colon cancer patients [29]. Moreover, Jin *et al.* confirmed that overexpression of Pim-1 associated with poor prognosis of non-small cell lung cancer [21]. Our findings corroborate the findings of these studies, yet more studies will be needed to define mechanisms of Pim-1 expression and function in ACC.

## Conclusion

The present study demonstrates that Pim-1 is important in cell proliferation, apoptosis, cell cycle, and invasion in both SACC-83 and SACC-LM cell lines. Pim-1 and RUNX3 levels were negative relevant and significantly associated with T-stage and nerve invasion in the ACC tissues. This study approves the oncogenic role of Pim-1 in ACC. The findings also suggest that Pim-1 may serve as a neoteric therapeutic target and potential prognostic marker for ACC cancer.
