# Correction: Pim-1 acts as an oncogene in human salivary gland adenoid cystic carcinoma

**DOI:** 10.1186/s13046-022-02509-9

**Published:** 2022-10-14

**Authors:** Xin Zhu, Jia-jie Xu, Si-si Hu, Jian-guo Feng, Lie-hao Jiang, Xiu-xiu Hou, Jun Cao, Jing Han, Zhi-qiang Ling, Ming-hua Ge

**Affiliations:** 1 Zhejiang Cancer Research Institute, Zhejiang Cancer Hospital, Hangzhou, 310022 China; 2 Department of Head and Neck Surgery, Zhejiang Cancer Hospital, Hangzhou, 310022 China

**Correction: J Exp Clin Cancer Res 33, 114 (2014)**

**https://doi.org/10.1186/s13046-014-0114-5**

Following publication of the original article [1], the author identified an error in Figs. 6 and 9. The defined scale bars were provided.

**Fig. 6:**
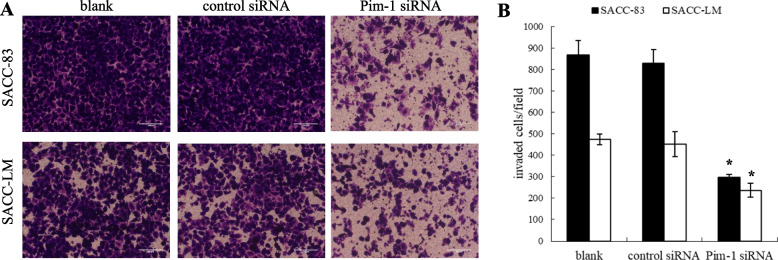
Suppressive effect of Pim-1 siRNA on the cell invasion in SACC cells. **A**. Crystal violet staining images of invasive SACC-83 and SACC-LM cells after Pim-1 siRNA transfection for 72 h. **B**. Quantification of the number of invaded SACC-83/SACC-LM in control siRNA and Pim-1 siRNA groups, respectively. Results were shownas mean ± SD. p < 0.05, *Pim-1 siRNA group compared with control siRNA group

**Fig. 9:**
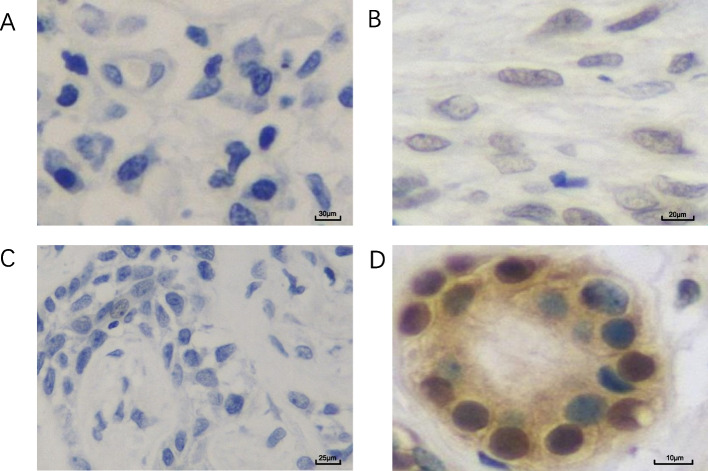
Immunohistochemical (IHC) staining of Pim-1 and RUNX3 in ACC. **A**. Negative Pim-IHC staining in ACC. **B**. Positive Pim-1 IHC staining in ACC. **C**. Negative RUNX3 IHC staining in ACC. **D**. Positive RUNX3 IHC staining in ACC. Magnificant factor: ×400.

The authors state that the image in Fig. 9C had been taken from the negative Pim-1 staining sample group in error. The corrected figure displays a representative image from the negative RUNX3 staining group.

This correction does not change the result, interpretation, and conclusions of the study.
